# Impact of a combined care ambulatory and home-based aerobic exercise program using wearables on anxiety and depression in patients with metabolic syndrome

**DOI:** 10.3389/fcvm.2026.1798233

**Published:** 2026-03-12

**Authors:** Jurate Zupkauskiene, Ieva Lauceviciene, Petras Navickas, Aleksandras Laucevicius

**Affiliations:** 1 Clinic of Cardiac and Vascular Diseases, Institute of Clinical Medicine, Faculty of Medicine, Vilnius University, Vilnius, Lithuania; 2 Department of Rehabilitation, Physical and Sports Medicine, Institute of Health Sciences, Faculty of Medicine, Vilnius University, Vilnius, Lithuania; 3 State Research Institute Center for Innovative Medicine, Vilnius, Lithuania

**Keywords:** aerobic exercise, anxiety, cardiometabolic risk, depression, home-based exercise, metabolic syndrome, physical activity, remote monitoring

## Abstract

**Background:**

Anxiety and depressive symptoms are more common in individuals with metabolic syndrome (MetS), which is characterized by abdominal obesity, subclinical atherosclerosis, and impaired glucose metabolism. Although aerobic exercise training (aET) with direct medical staff supervision improves cardiometabolic health, its implementation remains limited in this population. Technology-assisted physical activity (PA) interventions may enhance adherence, but their impact on mental health in MetS is not well established. Therefore, the aim of this study was to evaluate whether a supervised ambulatory aET program followed by a device-supported home-based aET program improves anxiety and depression levels in individuals with MetS.

**Methods:**

In this prospective study (NCT05592704), 132 adults with MetS (mean age 52.4 ± 6.3 years; 54.6% female) completed a 2-month supervised ambulatory aET program (40 min, 5 days/week). Participants were then randomized to: (1) a control group receiving only standard PA recommendations for 6 months, or (2) a intervention group receiving an individualized 6-month home-based aET program supported by the dedicated smartphone application and a Polar H10 heart-rate monitor. Anxiety and depression were assessed at baseline, after 2 and 8 months using the Hospital Anxiety and Depression Scale.

**Results:**

Linear Mixed Model analysis revealed significant Group × Time interactions for anxiety (*p* = 0.020) and depression (*p* = 0.049) after the 8-month combined care aEP. While groups remained comparable following the initial 2-month supervised ambulatory aET program (anxiety: *p* = 0.461; depression: *p* = 0.174), significant divergence occurred during the 6-month home-based aEP phase. By month 8, the intervention group achieved significantly lower scores than the control group for both anxiety (*p* = 0.005) and depression (*p* = 0.018). Notably, the intervention group demonstrated continued symptom reduction during the home-based phase (anxiety *Δ* = −1.09, −21.2%; depression *Δ* = −0.23, −9.7%), whereas the control group experienced symptomatic regression (anxiety *Δ* =  + 0.18, +4.2%; depression *Δ* =  + 0.21, +9.0%).

**Conclusion:**

A 2-month supervised ambulatory aET program followed by a 6-month home-based aET program with remote supervision using wearables significantly reduced both anxiety and depression levels in individuals with MetS. These findings demonstrate that combined care aET models not only initiate symptom reduction but also facilitate the long-term maintenance of mental health gains, highlighting the potential of technology-assisted interventions to enhance psychological outcomes in this population.

## 1 Introduction

Metabolic syndrome (MetS), a cluster of interrelated cardiovascular risk factors, affects nearly one third of the global adult population (1, 2). In 2023, MetS was estimated to affect approximately 1.54 billion adults worldwide, and its prevalence continues to rise (2). Compared with the general population, individuals with MetS have a higher prevalence of anxiety and depressive disorders (3, 4). Among middle-aged patients with MetS, depressive disorders have been reported in up to 25% of cases, while anxiety disorders may affect up to 30% (3). Consequently, the psychological aspects of MetS have been extensively examined in recent studies. Numerous studies have demonstrated a significant association between depressive or anxiety symptoms and key components of MetS, including abdominal obesity, elevated blood pressure (BP), and altered glucose and lipid metabolism (5–12).

Some studies report that MetS is more strongly associated with depressive symptoms than with anxiety (13, 14), whereas a recent study found no significant link between anxiety, depression, and obesity (15). Despite these conflicting findings, most of the research indicates that both anxiety and depression are associated with MetS, and vice versa (4, 16, 17). Therefore, it is important to identify effective interventions to reduce anxiety and depression levels in individuals with MetS. Addressing psychological factors should be considered an integral component of MetS management for more effective healthcare outcomes (15).

Aerobic exercise training (aET) is a well-established and effective health intervention for patients with MetS and mental health disorders (18, 19). Supervised aerobic exercise programs (aEP) have been shown to positively affect cardiometabolic profiles, arterial stiffness, and cardiorespiratory fitness (CRF), as well as to improve health-related quality of life (20–23). Individualized aerobic training with direct medical staff supervision can positively influence mental health and reduce the level of depression in MetS patients (23). The beneficial effects of physical activity (PA) on depression and anxiety are likely mediated by a combination of various psychological, social, and neurophysiological mechanisms (24). However, individuals with MetS are often physically inactive, and supervised aET programs—typically conducted in ambulatory settings—tend to be short-term, not widely implemented, and often inaccessible to this population (24). Challenges remain in maintaining adequate PA levels after ambulatory aET programs for high-risk cardiometabolic populations. Specifically, it remains unclear how to optimally adapt home-based programs to sustain behavioral changes and what specific duration is required to achieve permanent physiological and behavioral adaptations.

Digital health solutions can effectively promote PA among individuals with chronic diseases, while making such interventions more widely available and cost-efficient (25). There is growing evidence that combining wearable PA tracking devices with professional counselling can promote higher daily PA levels among patients with cardiometabolic disease (26, 27). Moreover, integrating wearable PA monitoring devices with dedicated smartphone applications may serve as an effective motivational tool, encouraging individuals with MetS to engage in regular PA (28, 29).

Compared with conventional unsupervised home-based aEP, interventions incorporating remote supervision and real-time heart rate (HR) monitoring are associated with higher adherence, improved precision in achieving prescribed exercise intensity, and superior physiological outcomes (30, 31). In patients with cardiovascular disease, home-based exercise supported by real-time HR monitoring allows most training sessions to be conducted within target HR zones and leads to significant gains in physical capacity (32). Moreover, in cardiac patients remote supervision integrated with digital monitoring and interactive feedback during home-based aEP is associated with improvements in anxiety and depression symptoms compared with unsupervised approaches (33). Therefore, we hypothesized that the program consisting of the outpatient ambulatory aEP followed by the additional electrocardiogram (ECG) targeted HR home-based training could improve anxiety and depression symptoms even more than the aEP without ECG HR targeting during home-based training. However, this approach remains insufficiently studied. To the best of our knowledge, there are no studies assessing changes in anxiety and depression symptoms under such a combination of PA interventions in people with MetS.

The objective of this prospective study was to evaluate changes in anxiety and depression levels by comparing two PA care models in patients with MetS: (1) the conventional model, in which, after completing an ambulatory aET program under direct medical supervision, participants received only standard PA recommendations for home-based training; and (2) the combined care model, in which participants, after the same ambulatory aET program, received both standard PA recommendations and a structured home-based aET program supported by smart wearable devices.

## 2 Materials and methods

### 2.1 Study design and population

This prospective study was conducted from 2017 to 2019 at the InMedica outpatient clinic, Lithuania, and approved by the Lithuanian Bioethics Committee (protocol No. PFAEV53; approval No. L-17-05/1; 7 July 2017). Written informed consent was obtained from all participants, and the study was registered at *ClinicalTrials.gov* (NCT05592704).

Participants were recruited from the Lithuanian High Cardiovascular Risk (LitHiR) program (34, 35). Eligible men (40–55 years) and women (50–65 years) met the NCEP ATP III (1) diagnostic criteria for MetS (≥3 of 5 components: abdominal obesity, elevated BP, impaired fasting glucose, hypertriglyceridemia, or low high-density lipoprotein cholesterol level). Participants with established cardiovascular disease, severe heart failure, or uncontrolled hypertension were excluded from the study.

**Figure 1:**
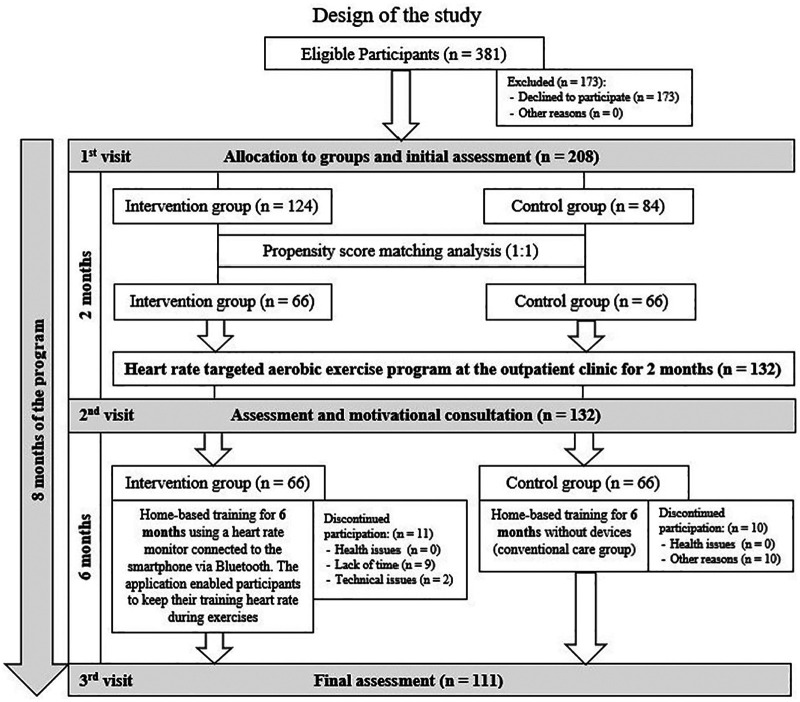
Flowchart of the study enrollment and evaluation after propensity score matching analysis.

A total of 208 individuals were randomly assigned to the intervention (combined care) or control (conventional care) group; allocation was not blinded. After 1:1 propensity score matching, 132 participants (66 per group) were included in the final analysis. The flowchart of the study enrollment and evaluation is presented in Figure 1.

### 2.2 Clinical assessment

Participants were evaluated at baseline (1st visit), after 2 months (2nd visit), and after 8 months (3rd visit). Anthropometric parameters [body mass index (BMI), waist circumference], BP, HR, and fasting blood samples for glucose and lipids were collected between 7:00 a.m. and 12:00 p.m. after an overnight fast, caffeine restriction, and at least 2 h after smoking. BP was measured in the seated position using validated oscillometric devices according to ESC/ESH 2018 guidelines (36). Arterial hypertension was defined as systolic BP ≥140 mmHg and/or diastolic BP ≥90 mmHg, or the use of antihypertensive drugs. Obesity and overweight were defined as BMI ≥30 kg/m^2^ and 25–29.9 kg/m^2^, respectively (36). Smoking status was obtained from medical records.

We evaluated CRF in all participants using an incremental cardiopulmonary exercise test (CPET) on a cycle ergometer at baseline, after 2 months, and after 8 months (37). The protocol started with 2 min of unloaded cycling, followed by a continuous ramp increase in workload (15–30 W/min) until volitional exhaustion or clinical termination. Following the American College of Sports Medicine (ACSM) guidelines, tests were terminated for clinical safety markers or volitional exhaustion, defined as a participant request to stop or the Borg Rating of Perceived Exertion (RPE) score ≥17 (38, 39). Each test lasted approximately 8–12 min, with continuous ECG and BP monitoring.

Gas exchange parameters were analyzed using a metabolic cart (Sensormedics Vmax ENCORE229, USA). The first ventilatory threshold (VT1) and the second ventilatory threshold (VT2) were determined using the V-slope and ventilatory equivalents methods (37, 40, 41). VT1 was identified as the point of the first disproportionate increase in VCO2 (carbon dioxide output) relative to VO2 (oxygen uptake). VT2 (the respiratory compensation point) was defined as the point where both VE/VO2 and VE/VCO2 (where VE is minute ventilation) showed a simultaneous increase (37, 40, 41).

The HR corresponding to VT1 was designated as the lower limit of training intensity, while the HR at the (VT2 represented the upper limit (37, 40, 41). These thresholds were used to individualize and monitor aerobic training intensity during the 6-month home-based phase.

CRF was expressed as maximal oxygen uptake (VO2 max, mL/kg/min) and metabolic equivalents (METs) achieved (37, 41). In this study, VO2max was defined as the highest VO2 value attained during a symptom-limited CPET where the participant reached the Respiratory Compensation Point (VT2)—identified by a systemic increase in the VE/VCO2 ratio—in combination with a Borg RPE score ≥17 (39). These criteria were met by all participants included in the final analysis, ensuring that the results reflect the maximal aerobic capacity reached under clinical supervision.

### 2.3 Assessment of anxiety and depression levels

Anxiety and depression levels were measured using the Hospital Anxiety and Depression Scale (HADS), a validated self-report questionnaire widely used to assess and monitor these symptoms in medical settings. The Lithuanian version has been applied since 1991 (42) and is suitable for individuals with MetS (13, 14).

The HADS consists of 14 items—7 assessing anxiety and 7 assessing depression (43). Each item is rated on a 4-point Likert scale (0–3), yielding subscale scores ranging from 0 to 21 for both anxiety and depression. Scores of 8–10 indicate mild, 11–14 moderate, and 15–21 severe anxiety or depression. Participants were instructed to complete the questionnaire quickly, as immediate responses are considered to best reflect their current emotional state (44).

### 2.4 Ambulatory aerobic exercise program

The study methodology has been previously described in detail (45). In brief, a 2-month ambulatory aEP was conducted at the outpatient clinic under the supervision of a rehabilitation specialist and cardiologist. All participants completed individualized, HR-targeted training on a cycle ergometer for 30–40 min/day, 5 days/week (40 sessions in total), following established aerobic exercise guidelines (37, 41).

Each session included a 10-min warm-up, a main training phase, and a 10-min cool-down. Warm-up began at 25 W, increasing to the prescribed HR, which was maintained during training and gradually reduced during cool-down. Exercise intensity corresponded to the HR at the first ventilatory threshold (VT1) during the first 2 weeks and was increased to the second ventilatory threshold (VT2) from week 3 onward. Real-time HR and workload were controlled using the Ergoline Rehabilitation System-2 (Ergoline GmbH, Germany). Pedalling cadence was set at 60 rpm. Training intensity was monitored using real-time HR to ensure participants remained between VT1 and VT2, supplemented by the Borg RPE scale (6–20) to track perceived exertion (39).

### 2.5 Motivational consultation and home-based aerobic exercise training

After completing the 2-month ambulatory aEP, all participants attended a 30-minute motivational consultation led by a rehabilitation specialist and a cardiologist. They received individualized guidance on cardiovascular risk reduction and home-based PA following standard recommendations (21, 46). The intervention and control group participants were encouraged to perform home-based aerobic exercises over the next 6 months at a similar frequency (30 min/day, 5 times/week).

During the motivational consultation, only the intervention group subjects received smartphones preloaded with the study application and paired with the Polar H10 HR monitor. Supervisors preconfigured devices by entering participant IDs and individual HR limits. Participants were fitted with the chest strap, instructed on device use, and asked to maintain HR within the target range for at least 50% of each workout time. All intervention group participants received written instructions, contact details for technical support.

During the 6-month home-based aET phase, participants in the intervention group performed workouts using assigned PA and HR self-monitoring devices. Participants were asked to send workout data via a smartphone application to the “Cardio training portal” as frequently as possible using Wi-Fi connectivity. This database enabled both the participant and the researcher to monitor the PA data accumulated in a computerized diary, allowing for a specialized protocol where subjects were actively involved in their progress and could discuss their data with the research team.

The researcher maintained constant contact with subjects primarily through telephone and email, with in-person clinic meetings scheduled only as necessary. This interactive support system provided continuous oversight and real-time feedback based on uploaded HR data to ensure participants from the intervention group maintained the prescribed training intensity within their individualized HR zones. It should be noted that the primary purpose of these wearable devices and the “Cardio training portal” was to enhance participant motivation and ensure adherence to the physiological quality of the exercise rather than to serve as an exhaustive system for collecting all PA data. Consequently, the monitoring focused on protocol fidelity and the maintenance of appropriate exercise intensity.

Subjects in the control group independently exercised for 6 months without using any PA self-monitoring devices, only based on the provided PA recommendations. Home-based exercises of the control group subjects were not registered in the study database. Also, the control group subjects did not receive phone calls, e-mails with exercise reminders.

We used a conventional care control group (standard PA recommendations) instead of a non-intervention group for ethical reasons, as participants had increased cardiometabolic risk. This design allowed us to isolate the added value of remote supervision and digital feedback over standard clinical advice, rather than simply measuring the effect of exercise itself.

### 2.6 Remote monitoring tools

**Figure 2:**
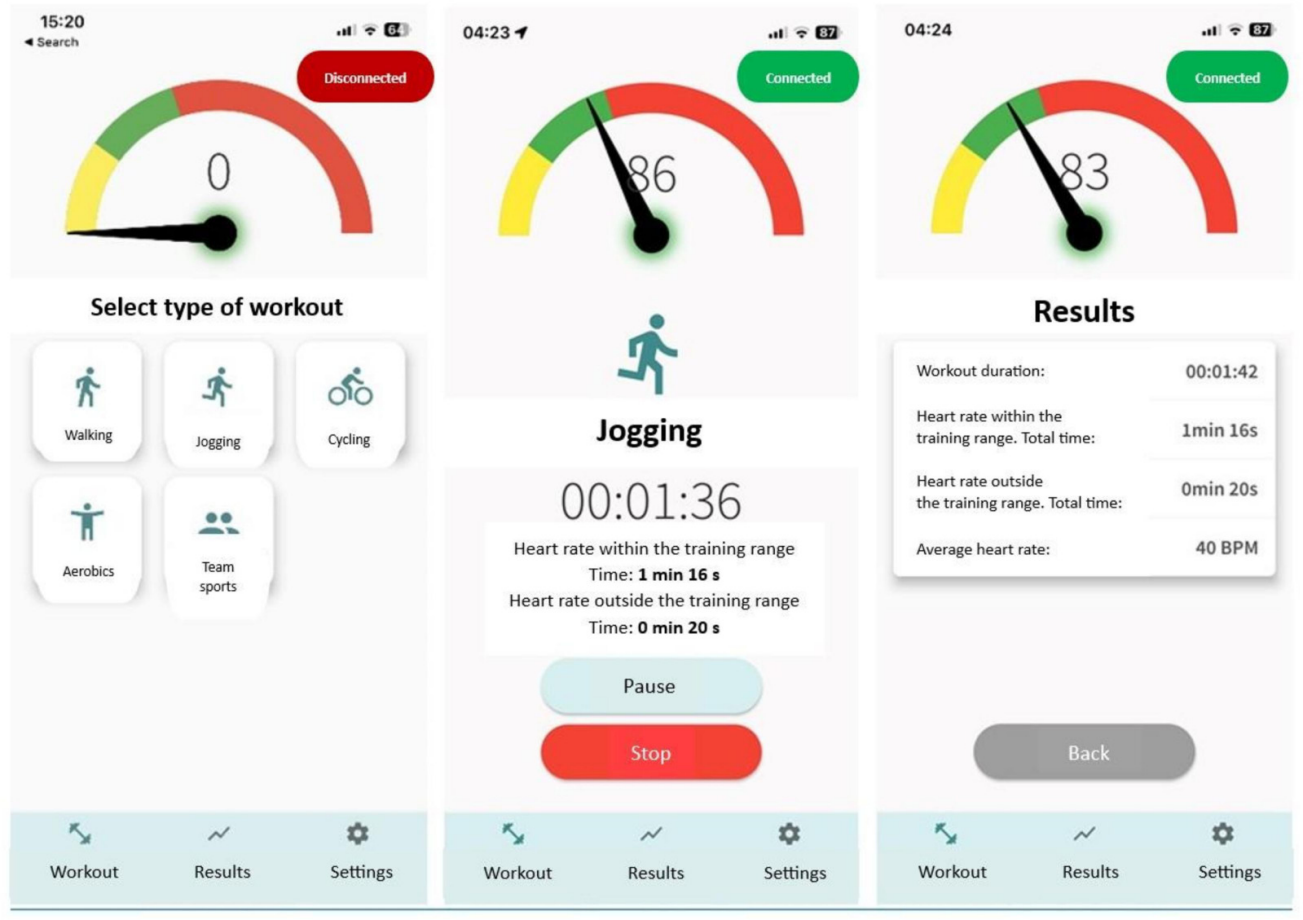
The main page and settings page of the study application with features (45).

Real-time HR monitoring during workouts was performed using the Polar H10 electrocardiogram-based chest strap (Polar Electro Oy, Kempele, Finland), rather than conventional trackers that rely on optical pulse-wave technology, which offer lower measurement precision. This elastic electrode-based device provides highly accurate HR measurements and is widely validated as a research reference tool (47–50). Each Polar H10 monitor was paired via Bluetooth with a Huawei Y5H smartphone (Huawei Technologies Co., Ltd, Shenzhen, China) to record HR data through a dedicated smartphone application, which was developed for this study in collaboration with information technology specialists using the Flutter platform (Android/iOS). The application allowed participants to self-monitor PA during the 6-month home-based training program. Key application features included the ability to select exercise type (e.g., aerobics, cycling, walking, jogging, team sports), track workout duration, and monitor HR in real time to ensure it remained within the target training range. Visual and audio feedback guided exercise intensity: yellow indicated HR below the target range, green within range, and red above range. Text and prompts were provided in Lithuanian, and auditory cues enabled participants to use the application without continuously monitoring the screen. Figure 2 presents the main interface and features of the application, as published previously (45).

### 2.7 Statistical analysis

Sample size and statistical power were determined *a priori* using G*Power (Version 3.1.9.6; Universität Kiel, Kiel, Germany). Prior studies indicate that aerobic exercise interventions typically yield medium effects on relevant outcomes (51–53). Accordingly, to detect a medium effect (Cohen's *d* = 0.5) in changes in anxiety and depression with 80% power at a 5% significance level, at least 64 participants per group were required. Effect sizes were classified as small (<0.20), moderate (0.20–0.79), or large (>0.80) (54). To enhance comparability and reduce bias, propensity score matching was applied to baseline data from the 208 participants initially enrolled. Matching was performed in XLSTAT (Version 2021.3.1) using the Mahalanobis distance method, considering baseline glucose and age as matching variables (caliper: 0.5 × SD; 1:1 matching; 95% CI; tolerance 0.001). After matching, each group included 66 participants, yielding a total of 132 for statistical analyses.

Data analysis was conducted using Jamovi (Version 2.2.5) (55). Normality was assessed with the Shapiro–Wilk test. Continuous variables are reported as mean ± standard deviation and categorical variables as counts and percentages. Baseline group comparisons used independent samples t-tests for normally distributed data and Mann–Whitney U-tests for non-normal data. Categorical variables were analyzed with *χ*^2^ or Fisher's exact tests.

Although the HADS is comprised of ordinal Likert items, the total subscale scores were treated as continuous interval data. This approach is consistent with established psychometric practices for validated aggregate scales and permits the use of Linear Mixed Models, which provide a more robust estimation of longitudinal changes compared to non-parametric alternatives. Internal consistency of the HADS subscales at baseline was assessed using Cronbach's alpha. At baseline, the HADS-Anxiety subscale demonstrated good internal consistency (Cronbach's *α* = 0.82), and the HADS-Depression subscale showed acceptable internal consistency (*α* = 0.72).

To assess the validity of the Missing at Random (MAR) assumption, a dropout analysis was performed. We compared baseline characteristics (demographics and primary outcomes) between participants who completed the final 8-month follow-up and those who did not, using independent samples t-tests and Chi-square tests. Differential attrition between the intervention and control groups was assessed using a Chi-square test.

To evaluate the effects of the intervention over time while accounting for participant attrition at the 6- and 8-month follow-ups, Linear Mixed Models (LMM) were estimated using the GAMLj module. This approach follows the Intention-to-Treat (ITT) principle by utilizing all available longitudinal observations through Restricted Maximum Likelihood (REML) estimation. The models included Group (Intervention vs. Control), Time (Baseline, 6-months, 8-months), and the Group × Time interaction as fixed effects. To account for the nested structure of the data and baseline variability, a random intercept for each participant was included. Degrees of freedom were calculated using the Satterthwaite approximation. *post-hoc* comparisons of the estimated marginal means were performed to identify specific points of divergence between groups. Statistical significance was defined as *p* < 0.05. Figure 1 illustrates the study enrolment and evaluation process following propensity score matching.

## 3 Results

### 3.1 Descriptive statistics

The final analysis included 132 individuals with MetS, of whom 54.6% were female and 60.6% were categorized as obese. Prior to enrolment, all participants reported engaging in less than the recommended level of moderate-intensity aerobic activity—less than 30 min/day, 5 days/week. Detailed baseline characteristics for the intention-to-treat population are provided in Table 1.

**Table 1:** Intention to treat: baseline characteristics of the participants (observed values, *N* = 132).

Parameter	Intervention group (*n* = 66)	Control group (*n* = 66)	*p* value between the groups
Age (years)	52.05 ± 6.15	52.83 ± 6.38	0.393
Sex			
Female (%)	34 (51.52)	38 (57.58)	0.484
Male (%)	32 (48.48)	28 (42.42)	
Height (m)	1.73 ± 0.10	1.70 ± 0.09	0.109
Weight (kg)	95.25 ± 15.34	87.76 ± 14.41	**0**.**006**
Waist circumference (cm)	104.55 ± 9.36	101.66 ± 9.23	0.086
Abdominal obesity (%)	58 (87.89)	58 (87.89)	1.000
BMI (kg/m^2^)	31.66 ± 4.01	30.59 ± 4.08	0.144
Normal BMI (%)	3 (4.55)	4 (6.06)	1.000
Overweight (%)	18 (27.27)	27 (40.91)	0.098
Obesity (%)	45 (68.18)	35 (53.03)	0.075
I degree (%)	29 (64.44)	25 (71.43)	0.479
II degree (%)	11 (24.44)	9 (25.71)	0.627
III degree (%)	5 (11.11)	1 (2.86)	0.210
Non smoking (%)	52 (78.79)	58 (87.88)	0.138
Smoking (%)	14 (21.21)	8 (12.12)	
< 10 cig./day	9 (64.29)	6 (75)	
> 10 cig./day	5 (35.71)	2 (25)	
Smoking cessation (%)	12 (18.18)	3 (4.55)	
Dyslipidemia (%)	63 (95.45)	66 (100)	0.244
Elevated fasting blood glucose (%)	29 (43.94)	36 (54.55)	0.223
Arterial hypertension (%)	41 (62.12)	62 (93.94)	**< 0.001**
Anxiety (scores)	5.95 ± 3.54	4.82 ± 2.78	0.069
Depression (scores)	3.53 ± 2.54	3.07 ± 2.59	0.248
Anxiety:			
0–7 scores (%)	46 (69.70)	53 (80.30)	
8–10 scores (%)	12 (18.18)	5 (7.58)	0.119
11–14 scores (%)	6 (9.09)	3 (4.55)	
15–21 scores (%)	1 (1.52)	0 (0)	
Depression:			
0–7 scores (%)	61 (92.42)	56 (84.85)	
8–10 scores (%)	3 (4.55)	5 (7.58)	0.482
11–14 scores (%)	1 (1.52)	0 (0)	
15–21 scores (%)	0 (0)	0 (0)	
VO2 max (mL/kg/min)	19.87 ± 3.98	21.27 ± 4.69	0.083
MET	5.63 ± 1.14	6.04 ± 1.30	0.073
Target training HR (bpm)	129.3 ± 11.92	128.5 ± 15.60	0.740

BMI, body mass index; cig./day, cigarettes per day; HR, heart rate; VO2 max, maximal oxygen consumption; MET, metabolic equivalent.

Data are presented as mean ± SD or in absolute numbers (*n*) and percentage in parentheses (%). *p* value in bold denotes a statistically significant difference (*p* < .05).

Participant flow and retention rates at 6 and 8 months are detailed in Figure 1. Drop-out analysis revealed no significant differences at baseline between completers and non-completers regarding age, BMI, or baseline HADS scores (all *p* > 0.05). Additionally, attrition rates were balanced between the intervention and control groups (*X*^2^ = 0.274, *p* = 0.60), suggesting that data were missing at random.

### 3.2 Changes in anxiety and depression levels during 8 months of combined care aEP

Anxiety and depression levels were assessed for both the intervention and control groups over the 8-month combined care aEP (Visit 1 to Visit 3) and during the 6-month home-based aEP phase separately (Visit 2 to Visit 3). Linear Mixed Model (LMM) analysis revealed a significant Group × Time interaction for both anxiety [*F* (2, 142.5) = 4.03, *p* = 0.020] and depression [*F* (2, 135.6) = 3.09, *p* = 0.049] (Table 2).

**Table 2:** Changes in anxiety and depression scores from baseline to 8-month follow-up: results of Intention-to-Treat analysis using Linear Mixed Models.

Outcome & time	Intervention (*n*) M (SE)	Control (n) M (SE)	Between-Group *p*
Anxiety score			
Baseline (V1)	(66) 6.06 (0.38)	(66) 4.75 (0.39)	0.244
2 months (V2)	(35) 5.13 (0.59)	(34) 4.30 (0.40)	0.461
8 months (V3)	(21) 4.04 (0.47)	(26) 4.48 (0.48)	**0**.**005**
*Δ* V1–V3 (8-months)	−2.01	−0.27	Interaction: **0.020**
*Δ* V2–V3 (6-months)	−1.09	+0.18	
Depression score			
Baseline (V1)	(66) 3.68 (0.28)	(66) 2.95 (0.28)	0.756
2 Months (V2)	(35) 2.37 (0.32)	(34) 2.33 (0.25)	0.174
8 Months (V3)	(21) 2.14 (0.25)	(26) 2.54 (0.28)	**0**.**018**
*Δ* V1–V3 (8-months)	−1.54	+0.41	Interaction: **0.049**
*Δ* V2–V3 (6-months)	−0.23	+0.21	

*n*, observed participants per visit; M (SE), Estimated Marginal Mean (Standard Error); V, visit. Analysis was performed using Linear Mixed Model analysis (LMM) with REML to account for missing data (Intention-to-Treat). Values are based on the total sample (*n* = 132). *Δ* indicates the change in mean score. Negative values represent symptom improvement. *p* value in bold denotes a statistically significant difference (*p* < .05).

Over the entire 8-month aEP, the intervention group demonstrated a significant reduction in estimated marginal mean (EMM) scores for both anxiety (Visit 1: 6.05 vs. Visit 3: 4.04) and depression levels (Visit 1: 3.68 vs. Visit 3: 2.14). In contrast, the control group showed smaller overall improvements in anxiety (Visit 1: 4.75 vs. Visit 3: 4.48) and depression (Visit 1: 2.95 vs. Visit 3: 2.54) (Table 2).

When examining the 6-month home-based aEP phase separately, a distinct divergence between groups was observed. While anxiety and depression scores were statistically comparable between groups at the end of the supervised phase (Visit 2: *p* = 0.461 for anxiety; *p* = 0.174 for depression), the intervention group achieved significantly lower scores by the final 8-month follow-up compared to the control group (*p* = 0.005 for anxiety and *p* = 0.018 for depression) (Table 2).

Specifically, during the home-based phase (Visit 2 to Visit 3), the intervention group's anxiety scores continued to improve (EMM: 5.13 to 4.04; change: −1.09; −21.2%), whereas the control group experienced a symptomatic regression (EMM: 4.30 to 4.48; change: +0.18; +4.2%). Similarly, for depression, the intervention group showed continued improvement (EMM: 2.37 to 2.14; change: −0.23; −9.7%), while the control group's scores increased (EMM: 2.33 to 2.54; change: +0.21; +9.0%). These divergent trajectories—where the intervention group maintained or built upon gains while the control group regressed—indicate that the home-based portal provided a therapeutic maintenance effect that was independent of the initial supervised stimulus (Table 2).

**Figure 3:**
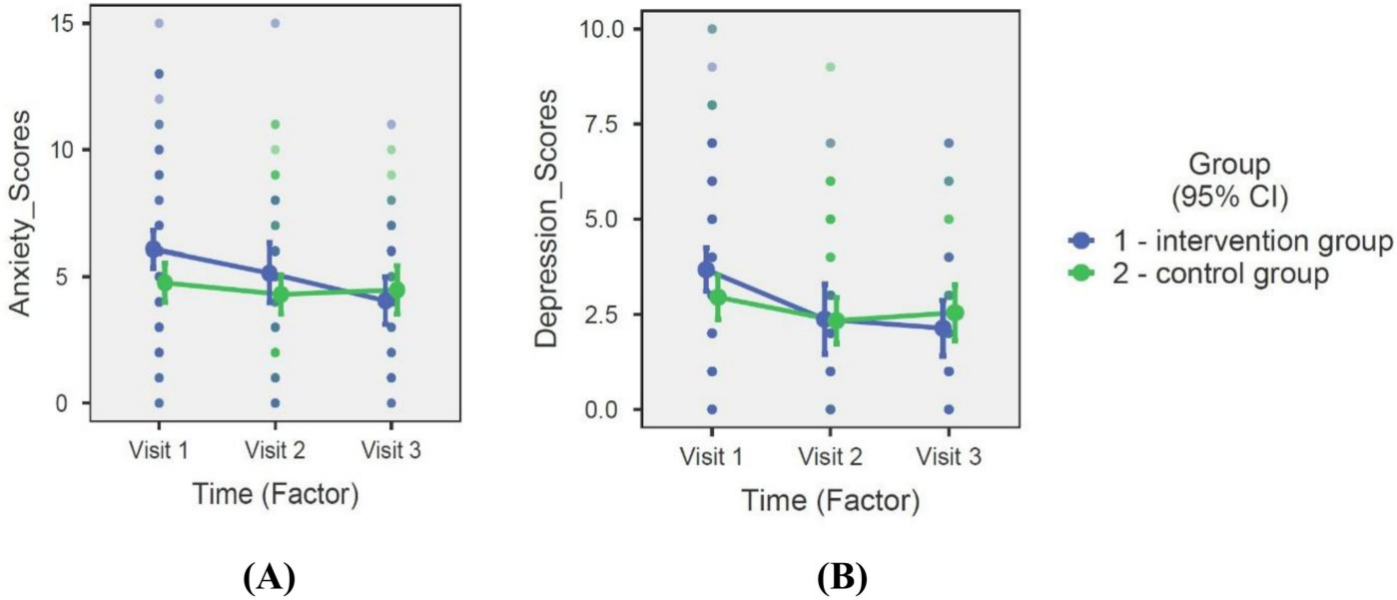
Logitudinal changes in anxiety and depression scores over the entire 8-month aerobic excercise program. Data are presented as Estimated Marginal Means; error bars represent the 95% Confidence Interval (CI). **(A)** HADS-Anxiety scores; **(B)** HADS-Depression scores. Significant Group × Time interacions (*p* < .05) were determined via Linear Mixed Model (LMM) analysis.

The results are illustrated in Figure 3.

## 4 Discussion

Our study evaluated the impact of an 8-month aET program that included 2 months of supervised ambulatory aET followed by 6 months of home-based aET on anxiety and depression levels in individuals with MetS. The primary finding is that this structured, combined-care model significantly improves mental health in this population. Linear Mixed Model (LMM) analysis revealed significant Group × Time interactions for both anxiety (*p* = 0.020) and depression (*p* = 0.049). While traditional exercise interventions typically emphasize short-term supervised training, our results demonstrate that a longer-term, 8-month commitment produces superior psychological profiles compared to standard care. These data suggest that the cumulative volume and extended duration of aerobic exercise are critical for stabilizing mental health and achieving clinically meaningful improvements in this high-risk population.

To our knowledge, no previous research has investigated a combined ambulatory and home-based aET program incorporating smart wearable devices to address symptoms of anxiety and depression in individuals with MetS. Existing evidence has largely focused on supervised ambulatory programs, primarily within type 2 diabetes populations, with varying results. For instance, Maharaj et al. reported significant improvements in both anxiety and depression following a 12-week treadmill-based intervention (44). Similarly, Silva et al. documented marked reductions in depression (57%) and anxiety (41%) using a 12-week supervised aquatic program guided by PA monitoring; however, the absence of a control group in that study limits the interpretability of those findings (56). Furthermore, the consistency of these psychological benefits remains a subject of debate. While Delevatti et al. observed improvements in quality-of-life domains, they detected no significant changes in depression scores (57). Similarly, Gilani et al. reported significant reductions in anxiety (*p* = 0.044) but failed to find a significant effect on depression after 12 weeks of combined aerobic and resistance training (58). Although several investigations suggest that supervised ambulatory aET can reduce depressive symptoms in type 2 diabetes, the broader evidence remains inconsistent, with psychosocial outcomes differing significantly across study designs (59–61).

Several studies have demonstrated that lifestyle interventions incorporating directly supervised aET can yield beneficial effects on anxiety and depression in overweight populations (62, 63). Nevertheless, evidence remains inconsistent. The OPTICARE XL randomized controlled trial demonstrated that a comprehensive 1-year cardiac rehabilitation program, including 3 months of combined endurance and resistance training, behavioral coaching, and a subsequent 9-month after-care phase, did not confer additional benefits for anxiety or depression compared with standard 6–12-week rehabilitation consisting of endurance training and cardiovascular lifestyle education (64). Both groups showed improvements over time, with no significant between-group differences (64).

Data on the psychoemotional impact of ambulatory aET specifically in individuals with MetS remain scarce. In a 12-week ambulatory aET intervention among women with MetS, Morga et al. reported substantial reductions in depressive symptoms (−37%) and perceived stress (−23%) (both *p* < 0.01), although the absence of a control group limits the interpretability of these findings (65). Our research group previously conducted a prospective study evaluating a 2-month supervised ambulatory aET program in individuals with MetS and demonstrated a significant reduction in depressive symptoms as measured by the HADS (*p* = 0.021) (23).

In our study, we extended the 2-month ambulatory induction by incorporating a 6-month home-based aET phase supported by remote monitoring. This combined approach resulted in significant 8-month reductions in both anxiety and depression levels in individuals with MetS. Following the 6-month home-based phase (Visit 2 to Visit 3), a distinct divergence was observed: the intervention group showed continued reductions in anxiety (−1.09 points; −21.2%) and depression (−0.23 points; −9.7%), whereas scores in the control group increased, reflecting a symptomatic regression (anxiety: +0.18 points; +4.2%; depression: +0.21 points; +9.0%).

While the isolated change within the intervention group during this home-based aET phase was a continuation of earlier gains, the contrasting trajectories—sustained improvement in the intervention group vs. regression in the control group—were the primary drivers of the significant Group × Time interactions observed at the study's conclusion (*p* = 0.020 for anxiety and *p* = 0.049 for depression). By Visit 3, the intervention group achieved significantly lower scores than the control group for both anxiety (*p* = 0.005) and depression (*p* = 0.018). These findings indicate that the home-based phase provided a critical maintenance effect, preventing the symptomatic relapse observed in the control group once supervised clinical contact was removed.

This ability to maintain and even build upon psychological gains stands in contrast to previous research. For instance, a 2023 pilot study in patients with MetS implemented 12 weeks of home-based aET with remote monitoring using similar devices; however, depression scores remained unchanged, and the study's interpretability was limited by the lack of a control group (66). Notably, in our study, participants utilized a dedicated smartphone application for PA self-monitoring alongside smart wearables, which may have facilitated the continued symptom decline observed during the home-based phase.

Our results contrast with those of Taylor et al., who reported no significant changes in anxiety or depression in an obese diabetic population following a 1-year electronic lifestyle program encouraging pedometer use (67). The divergence observed in our intervention group suggests that the effectiveness of technology-assisted interventions on psycho-emotional outcomes depends heavily on program structure and the level of interactivity. Specifically, the inclusion of a supervised “induction” phase (Visit 1 to Visit 2) followed by a seamlessly transitioned home phase may provide a more robust psychological stimulus than independent, pedometer-based programs alone.

Furthermore, while some evidence suggests that home-based aET with remote monitoring may not significantly impact anxiety or depression in cardiometabolic populations (68, 69), other technology-supported trials support our findings. For instance, mobile-app-based lifestyle programs, virtual-reality cycling, and intensive online lifestyle management have all demonstrated significant reductions in psychological distress. These disparate findings suggest that changes in anxiety and depression are strongly dependent on the specific design and delivery of the intervention (70–72). Programs that incorporate interactive, technology-supported communication between participants and researchers appear more effective in improving psycho-emotional outcomes in individuals with elevated cardiometabolic risk.

From a psychological perspective, exercise may provide social support, serve as a distraction from distress, and may alleviate anxiety and depression by enhancing self-esteem and perceived mastery (24). Specifically, remotely monitored home-based exercise enhances behavioral engagement and adherence, thereby increasing exercise dose and consistency—key determinants of sustained neurophysiological adaptations, including repeated upregulation of neurotrophins and attenuation of chronic stress responses (73). Regular exercise has been shown to modulate monoamine neurotransmitters (e.g., serotonin, dopamine, and norepinephrine) and regulate cortisol levels, induce the increase in irisin and Brain-Derived Neurotrophic Factor (BDNF) concentrations, thereby contributing to mood improvement and anxiety reduction (74, 75).

In our study, the 6-month home-based aET phase incorporated multiple interactive components, including a smartphone application providing real-time PA performance feedback, congratulatory and motivational messages, and access to the “Cardio Training Portal”, which enabled structured monitoring of cardiometabolic risk and PA (76). Participants in the intervention group were also able to contact the research team directly to address questions as needed. These features likely contributed to enhanced engagement and may partially explain the positive psycho-emotional trends observed in our intervention group.

Several limitations related to our study should be considered. First, the longitudinal nature of the 8-month program resulted in missing data due to participant attrition at scheduled visits or the incomplete return of psychosocial questionnaires. To address this and maintain the Intention-to-Treat (ITT) principle, we employed Linear Mixed Model (LMM) analysis with Restricted Maximum Likelihood (REML) estimation. Unlike complete-case analysis, LMM accounts for the dependency of repeated measures and utilizes all available data points, providing more robust and unbiased estimates of the treatment effect despite the presence of missing values. While LMM analysis revealed significant between-group differences at 8 months, the contrast was likely narrowed by our ethical decision to provide the control group with the initial 2-month supervised aET program and PA recommendations for home-based training. This active control setup may have led to an underestimation of the intervention's full effect size. Additionally, as the optimal duration for home-based exercise interventions is not yet established, further research is needed to determine if an 8-month period is the ideal timeframe for sustaining these psychological gains. We hypothesize that a more personalized approach—involving individually tailored ambulatory prescriptions and a continuation of home-based training with more regular, bi-directional feedback from medical staff—could further enhance exercise motivation and lead to even greater reductions in distress. Third, the study cohort was relatively homogeneous in terms of age and clinical characteristics, which may constrain the generalizability of these findings to broader or more diverse populations with MetS. Moreover, participants in the intervention group utilized wearable devices during the home-based aET primarily to increase exercise motivation and maintain appropriate intensity by targeting training HR zones. These devices were intended as a feedback tool for the subjects rather than a primary means of data collection. Consequently, a comparison of the total number of home-based workouts between the intervention and control groups was not analyzed in the present study. Despite these limitations, to our knowledge, this is the first study to examine changes in anxiety and depression in individuals with MetS following a personalized ambulatory aET program extended by a home-based phase incorporating ECG-based heart rate monitoring and smart-device–supported guidance.

## 5 Conclusions

Implementing a 2-month supervised ambulatory aET program followed by a 6-month home-based phase with remote wearable-based monitoring significantly reduced both anxiety and depression levels in individuals with MetS. While the supervised phase initiated these improvements, the subsequent home-based phase led to a significant divergence from the control group, ensuring the long-term maintenance of mental health gains. To our knowledge, these findings are among the first to demonstrate such sustained effects and provide novel evidence supporting the benefits of long-term, technology-assisted combined care aET for improving mental health in the MetS population.

## Data Availability

The original contributions presented in the study are included in the article/Supplementary Material, further inquiries can be directed to the corresponding author.
